# Radiation Induces Distinct Changes in Defined Subpopulations of Neural Stem and Progenitor Cells in the Adult Hippocampus

**DOI:** 10.3389/fnins.2018.01013

**Published:** 2019-01-09

**Authors:** Olga A. Mineyeva, Dmitri V. Bezriadnov, Alexander V. Kedrov, Alexander A. Lazutkin, Konstantin V. Anokhin, Grigori N. Enikolopov

**Affiliations:** ^1^Moscow Institute of Physics and Technology, Dolgoprudny, Russia; ^2^P. K. Anokhin Research Institute of Normal Physiology, Moscow, Russia; ^3^National Research Center “Kurchatov Institute,” Moscow, Russia; ^4^N.N. Burdenko Neurosurgery Institute, Moscow, Russia; ^5^Center for Developmental Genetics, Department of Anesthesiology, Stony Brook University, Stony Brook, NY, United States

**Keywords:** adult neurogenesis, stem cells, quiescent progenitors, gamma irradiation, nucleotide labeling

## Abstract

While irradiation can effectively treat brain tumors, this therapy also causes cognitive impairments, some of which may stem from the disruption of hippocampal neurogenesis. To study how radiation affects neurogenesis, we combine phenotyping of subpopulations of hippocampal neural stem and progenitor cells with double- and triple S-phase labeling paradigms. Using this approach, we reveal new features of division, survival, and differentiation of neural stem and progenitor cells after exposure to gamma radiation. We show that dividing neural stem cells, while susceptible to damage induced by gamma rays, are less vulnerable than their rapidly amplifying progeny. We also show that dividing stem and progenitor cells that survive irradiation are suppressed in their ability to replicate 0.5–1 day after the radiation exposure. Suppression of division is also observed for cells that entered the cell cycle after irradiation or were not in the S phase at the time of exposure. Determining the longer term effects of irradiation, we found that 2 months after exposure, radiation-induced suppression of division is partially relieved for both stem and progenitor cells, without evidence for compensatory symmetric divisions as a means to restore the normal level of neurogenesis. By that time, most mature young neurons, born 2–4 weeks after the irradiation, still bear the consequences of radiation exposure, unlike younger neurons undergoing early stages of differentiation without overt signs of deficient maturation. Later, 6 months after an exposure to 5 Gy, cell proliferation and neurogenesis are further impaired, though neural stem cells are still available in the niche, and their pool is preserved. Our results indicate that various subpopulations of stem and progenitor cells in the adult hippocampus have different susceptibility to gamma radiation, and that neurogenesis, even after a temporary restoration, is impaired in the long term after exposure to gamma rays. Our study provides a framework for investigating critical issues of neural stem cell maintenance, aging, interaction with their microenvironment, and post-irradiation therapy.

## Introduction

Radiation therapy with gamma rays is an indispensable tool for treating brain tumors in children and adults. While this irradiation can effectively eliminate cancer cells, it also induces profound cognitive impairments in up to 90% of patients undergoing whole-brain radiation therapy—a treatment used by more than 200,000 patients a year in the United States alone (Perry and Schmidt, 2006; Ahles et al., 2012; DeSantis et al., 2014; Rapp et al., 2015; Smart, 2017). These radiation-induced impairments include deficits in short- and long-term memory and learning, as well as increased anxiety and depression. The profile of irradiation-induced neuropsychological and behavioral changes raises the possibility that they may stem in part from disruptions of hippocampal neurogenesis, a critical factor in learning and memory, emotional state, and response to stress (Aimone et al., 2014; Christian et al., 2014; Abrous and Wojtowicz, 2015; Cameron and Glover, 2015; Kempermann et al., 2018).

If so, to mitigate irradiation’s harmful cognitive consequences, we must better understand its effects on hippocampal neurogenesis. In rodents and humans, new neurons are born in the dentate gyrus (DG), within a stem cell niche that harbors precursors to neuronal and glial cells (Seri et al., 2004; Knoth et al., 2010; Bonaguidi et al., 2011; Encinas et al., 2011; Spalding et al., 2013; Boldrini et al., 2018; Kempermann et al., 2018; Sorrells et al., 2018). These precursors divide to produce highly plastic young neurons that mediate experience-dependent changes in connectivity within the hippocampus and between the hippocampus, cortex, amygdala, and other brain regions (Vivar et al., 2012; Bergami et al., 2015; Vivar et al., 2016). Irradiation eliminates dividing neural precursors and causes inflammation and vascular damage in the brain (Monje et al., 2002; Mizumatsu et al., 2003; Monje et al., 2003; Rola et al., 2004a,b, 2005, 2008; Moravan et al., 2011; Allen et al., 2013, 2014; Sweet et al., 2014; Acharya et al., 2015). Indeed, the extent of irradiation-induced damage to neural precursors partially predicts the duration of neurogenic and cognitive deficiencies, indicating that these perturbations may be a significant cause of irradiation-induced cognitive impairments (Rola et al., 2004b; Achanta et al., 2009; Ji et al., 2014; Son et al., 2014). Thus, strategies for mitigating radiation’s cognitive side effects may require full or partial restoration of the neurogenic potential of the adult brain.

The neurogenic capacity of the hippocampus rests on the main class of neural precursors in the DG, commonly referred to as “multipotent neural stem cells” or, based on their morphology, “radial glia-like cells” (RGLs) (Kronenberg et al., 2003; Kempermann et al., 2004; Mignone et al., 2004; Seri et al., 2004; Encinas et al., 2006; Bonaguidi et al., 2011; Encinas et al., 2011; Shin et al., 2015; Urban et al., 2016; Pilz et al., 2018). RGL cells produce rapidly amplifying progeny (amplifying neural progenitors, ANPs) which, after a series of transitions, give rise to new hippocampal neurons (Kempermann et al., 2004; Encinas et al., 2011; Enikolopov et al., 2015). Previously, we have shown that new DG neurons are generated at the expense of RGLs: while mostly quiescent, RGL stem cells initiate a series of asymmetric neurogenic divisions upon activation and then leave the stem cell pool via conversion into astrocytes (Encinas et al., 2011). The rate of stem cell pool expenditure can be affected by a variety of factors, including damage to stem cells, their excessive activation and recruitment, reduced output, premature senescence, and inflammatory milieu. Later, these changes in the stem cell pool may be translated into changes in the number or in the connectivity of new neurons and eventually manifest in behavioral and cognitive performance (as in patients treated with radiation).

Exposure to radiation can potentially affect neural stem cells at several points of vulnerability, in addition to destroying actively dividing RGLs and their progeny: radiation may augment or suppress cell cycle activation of RGL cells, reduce the reserve of quiescent RGLs, alter stem cell output, induce premature senescence of stem cells or their microenvironment, or induce neuroinflammation in their niche. These effects may be compounded by the impact of radiation on the connections established or maintained by the newborn neurons. While the mechanisms of radiation action on dividing progenitor cells in the hippocampus have been thoroughly studied (Monje et al., 2002; Mizumatsu et al., 2003; Rola et al., 2004a, 2005, 2008), surprisingly little is known about the effect of therapeutic irradiation on quiescent stem cells. In part, gaps in understanding the action of radiation on neural stem cells are due to the limitations of conventional cell cycle analysis methods.

Here, we use a combination of genetic marking of stem and progenitor cells with differential tagging of newborn DNA to determine the immediate and long-term effects of exposing stem and progenitor cells to moderate doses of gamma rays. Our results demonstrate the vulnerability of dividing and non-dividing neural stem cells to radiation, these cells’ potential to restore their radiation-affected function, and the lingering effects of radiation on maturation of young neurons and productivity of stem cells.

## Results

### Acute Effects of Radiation on Neural Progenitors: 1 Day Post-exposure

**FIGURE 1:**
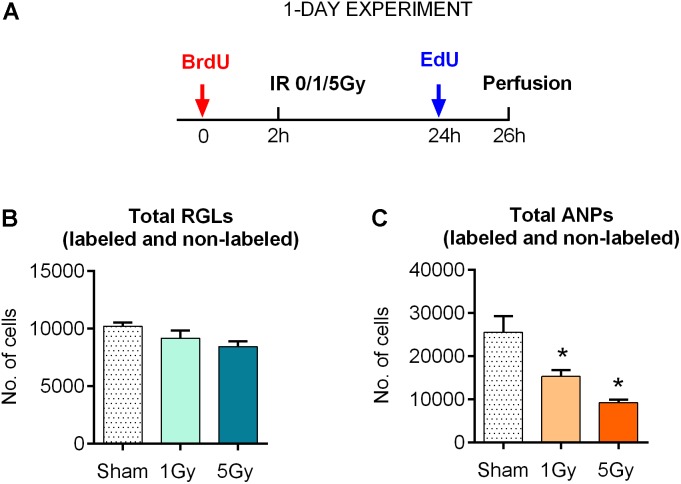
Response of neural progenitors to gamma radiation (1-day experiment). **(A)** Experimental design. **(B)** Response to radiation of radial glia-like neural stem cells (RGLs) 24 h post-exposure to 0, 1, or 5 Gy of gamma rays. **(C)** Same for amplifying neural progenitors (ANPs). ^∗^*p* < 0.05, a comparison with sham group, Dunnett’s multiple comparison test (see Supplementary Table S1 for detailed statistics). Bars show means and standard errors. *N* = 4 mice were used in 0 Gy group, *n* = 5 in 1 Gy group, and *n* = 4 in 5 Gy group.

We first addressed the acute effects of gamma rays by measuring survival and proliferation of neural stem and progenitor cells in the DG 24 h after exposure to gamma radiation. To differentiate between various classes of neuronal precursors, we employed Nestin-GFP reporter mice, in which GFP expression marks RGLs and their early progeny, the ANPs (Mignone et al., 2004; Enikolopov et al., 2015; Mignone et al., 2016). These reporter-expressing RGL cells include neural stem cells defined as type-1 cells (Kronenberg et al., 2003; Kempermann et al., 2004), radial astrocytes (Seri et al., 2001, 2004), and quiescent neural progenitors, QNPs (Mignone et al., 2004; Encinas et al., 2006). While most RGLs are quiescent (1–1.5% of RGLs are pulse-labeled with thymidine analogs), their ANP progeny are actively dividing [10–30% can be pulse-labeled (Encinas et al., 2006, 2011)]. To increase the resolution of our analysis and assess particular cohorts of stem and progenitor cells, we combined cell phenotyping with double S phase labeling of dividing cells using thymidine analogs: a halogenated nucleotide 5-bromo-2′-deoxyuridine (BrdU) and a terminal alkyne-bearing nucleotide 5-ethynil-2′-deoxyuridine (EdU). Mice were injected with BrdU and 2 h later exposed to gamma rays at the doses of 1 and 5 Gy, or 0 Gy (sham-treated control group). The mice were also injected with EdU 24 h after the BrdU injection and were sacrificed 2 h later (Figure 1A); by that time damaged cells of the niche exposed to radiation are expected to be removed by apoptosis (Li et al., 2016), facilitating the analysis. We were able to unambiguously reveal BrdU and EdU residues incorporated into newly synthesized DNA using an antibody and a fluorescent azide, respectively (Podgorny et al., 2018a,b). We phenotyped GFP-expressing cells as RGLs if they possessed a radial process positive for glial acidic fibrillary protein (GFAP) non-branching within at least a third of the granular layer, or as ANPs if they lacked a radial process and had round or oval morphology (Figure 3); we further identified additional classes of neural progenitors using markers such as doublecortin (DCX) and taking their morphology into account (Encinas and Enikolopov, 2008). By combining double-birthdating with cell phenotyping, we were able to increase the precision of our analysis and measure cell proliferation, cell cycle reentry, and survival of defined subpopulations, in parallel.

With these tools, we first examined the impact of gamma rays on the entire pool of stem (RGL) cells. We did not find a statistically significant decrease in the total number of RGL cells 24 h after exposure to 1 or 5 Gy (10% decrease, *P* = 0.33, and 17% decrease, *P* = 0.09 for 1 and 5 Gy, respectively; the CI and ANOVA values for this and the following experiments are presented in Supplementary Table S1 and (Figure 1B). These results are compatible with the observation that only a small fraction (1–2%) of RGL cells are in the S phase at a given time, and even the loss of the entire dividing subpopulation should not noticeably change the overall number of RGL cells in the DG. These results suggest that non-dividing RGL are resistant to 1–5 Gy of gamma irradiation. In contrast, the total number of ANPs decreased by 40% after 1 Gy (*P* = 0.024) and 64% after 5 Gy (*P* = 0.002), compatible with the cycling status of the majority of ANP cells (Figure 1C and Supplementary Table S1).

Next, we investigated radiation-induced changes in defined subclasses of progenitors by quantifying RGL and ANP cells carrying different labels and their combinations. We analyzed the following parameters:

(a) the number of BrdU^+^ cells, which correspond to the cells in S phase at the time of BrdU injection [the bioavailability of BrdU and other thymidine analogs may not exceed 1 h, therefore, this analysis represents a snapshot of the division status at the time of label injection (Mandyam et al., 2007; Kuhn et al., 2016)];
(b) the number of EdU^+^ cells, which correspond to the cells in S phase 2 h before the analysis (and therefore 22 h after irradiation and 24 h after BrdU injection);
(c) the number of double-labeled BrdU^+^EdU^+^ cells, which correspond to the cells that were in S phase both at the time of BrdU injection and again at the time of EdU injection;
(d) finally, the number of BrdU^-^EdU^+^ (i.e., EdU^*only*^) cells, which correspond to the cells that were in S phase at the time of EdU injection, but not at the time of BrdU injection.

**FIGURE 2:**
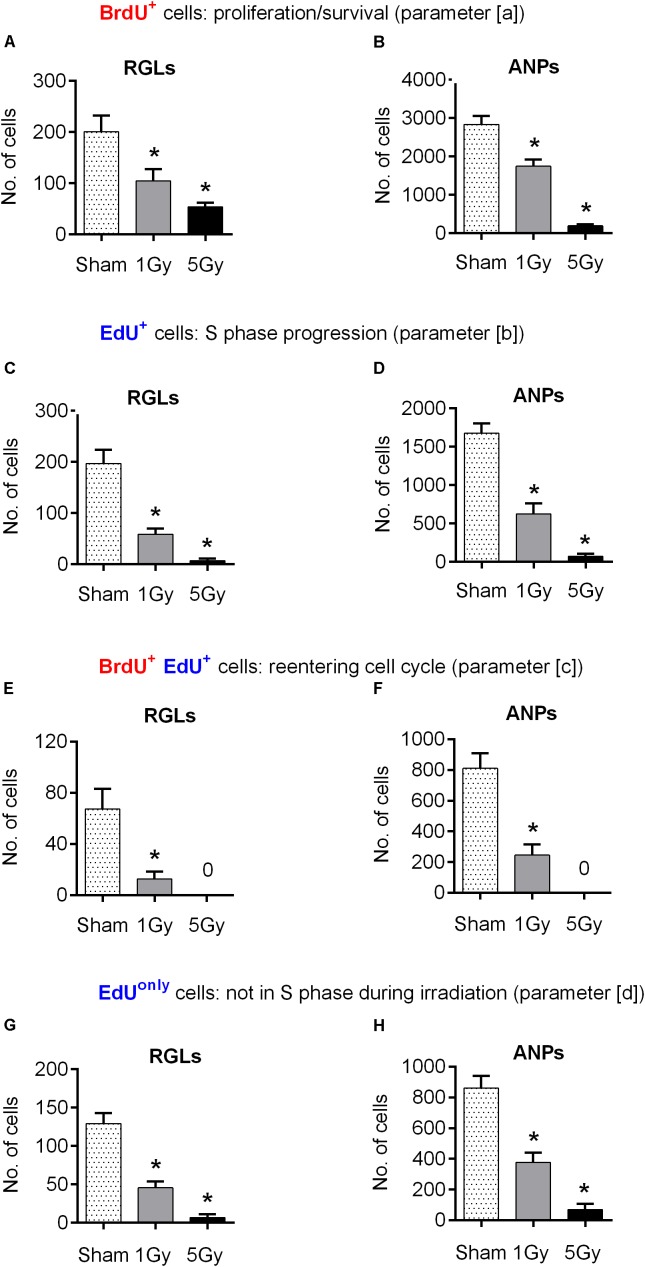
Progenitor cell proliferation at 24 h post-exposure to 0, 1, or 5 Gy (1-day experiment—scheme in Figure 1A). **(A, B)** BrdU^+^ RGL **(A)** and ANP **(B)** cells, representing cells that were in S phase by the time of irradiation (parameter [a]; see text for details). **(C, D)** EdU^+^ RGL **(C)** and ANP **(D)** cells, representing cells that were in S phase by the time of perfusion (parameter [b]). **(E, F)** BrdU^+^ EdU^+^ double-labeled RGL **(E)** and ANP **(F)** cells, representing cells that reentered the cell cycle, i.e., that were in S phase by the time of irradiation and also by the time of perfusion (parameter [c]). **(G, H)** EdU^*only*^, i.e., BrdU^–^EdU^+^ RGL **(G)** and ANP **(H)** cells, representing cells that were in S phase by the time of perfusion but not by the time of irradiation (parameter [d]). ^∗^*p* < 0.05, a comparison with sham group, Dunnett’s multiple comparison test for all cell groups after one-way ANOVA, multiple *t*-tests with Holm–Sidak multiple comparison method for BrdU^+^EdU^+^ cells (see Supplementary Table S1 for detailed statistics). Bars show means and standard errors. *N* = 4 mice were used in 0 Gy group, *n* = 5 in 1 Gy group, and *n* = 4 in 5 Gy group. Examples of cells counted are shown in Figure 3.

We began by tracking changes in the number of BrdU-labeled precursors (parameter [a] above), most of which were still in S phase at the time of irradiation [with the S phase duration of ∼8–10 h (Encinas et al., 2011; Podgorny et al., 2018b)]. We found that exposure to 1 Gy led to a 48% decrease in the number of BrdU-labeled RGL (*p* = 0.03) and a 42% decrease of BrdU-labeled ANPs (*p* = 0.0004) compared to the sham group (Figures 2A,B, 4 and Supplementary Table S1). Exposure to 5 Gy resulted in 73% decrease in BrdU-labeled RGL (*p* = 0.003) and 94% decrease of BrdU-labeled ANPs (*p* = 0.0001) (Figures 2A,B, 4 and Supplementary Table S1), with ANOVA showing a large main effect of gamma irradiation for both cell types and two-way ANOVA showing interaction between cell type and dose factors [*F*(2,10) = 84, *p* < 0.0001].

**FIGURE 3:**
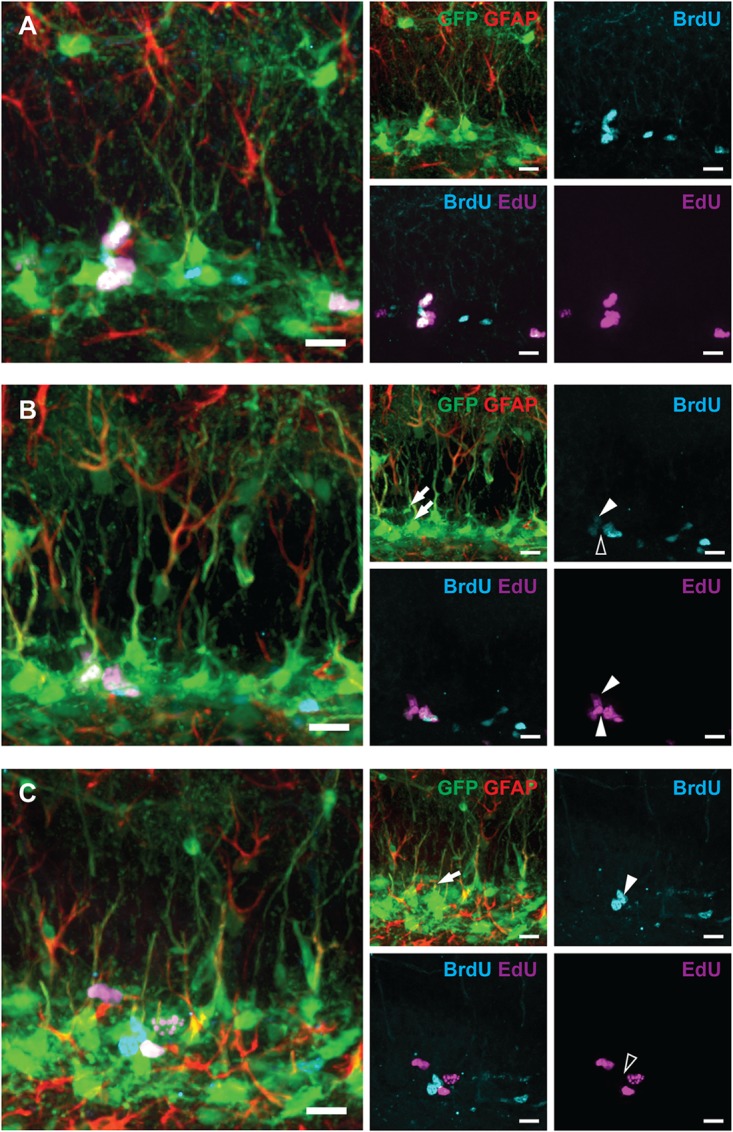
Examples of labeled RGLs and ANPs analyzed in Figure 2 (1-day experiment—scheme in Figure 1A). **(A)** BrdU^*only*^, EdU^*only*^, and BrdU^+^EdU^+^ labeled ANPs. **(B)** EdU^*only*^ labeled RGL [lower arrow on GFAP and GFP channels’ overlay, lower arrowhead (white) in EdU channel, and same position with no labeling shown with blank arrowhead in BrdU channel], BrdU^+^EdU^+^ labeled RGL [upper arrow in GFP and GFAP channels’ overlay, upper arrowhead (white) in BrdU channel, and upper arrowhead (white) in EdU channel], other labeled cells represent ANPs. **(C)** A BrdU^*only*^ labeled RGL (arrow in GFAP and GFP channels’ overlay, arrowhead in BrdU channel, and same position with no labeling shown with blank arrowhead in EdU channel), other labeled cells represent ANPs. Scale bars show 20 μm.

**FIGURE 4:**
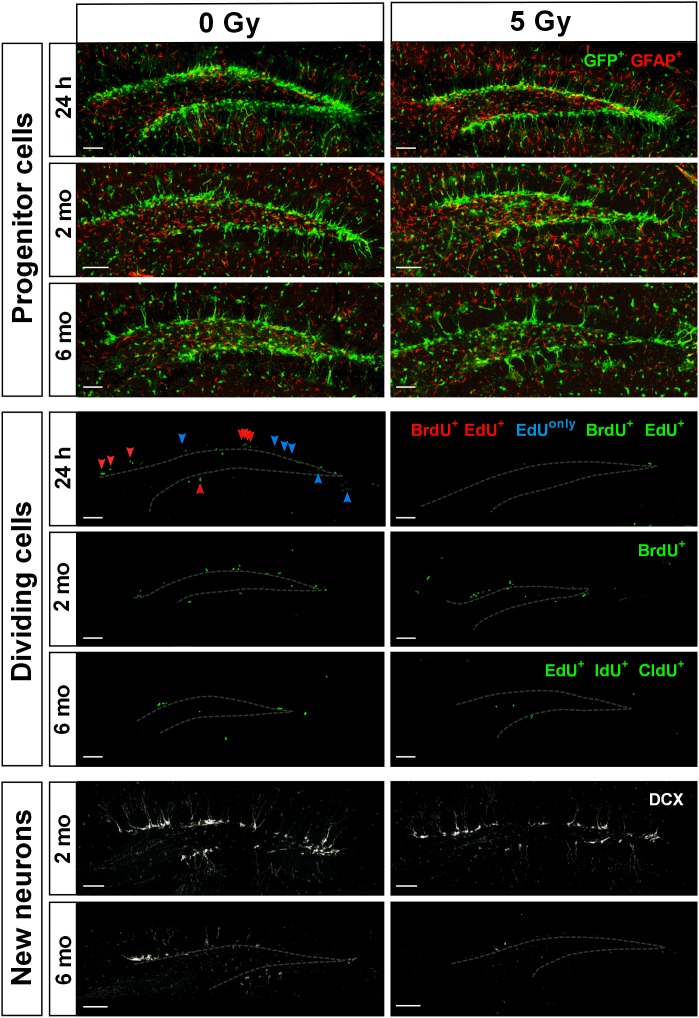
Maintenance of SGZ neural progenitors at 1 day, 2 months, and 6 months post-irradiation with 5 Gy dose. Upper three rows show Nestin-GFP-positive progenitors in the SGZ. Next three rows show dividing cells labeled according to the protocols described in Figures 1A, 5A, 7A. Green color is used for all injected nucleotides. In the fourth row, BrdU^+^ cells representing cells that were in S phase by the time of irradiation (parameter [a]; see text for details); EdU^+^ cells representing cells that were in S phase by the time of perfusion (parameter [b]); BrdU^+^EdU^+^ double-labeled cells (red arrowheads) representing cells that were in S phase by the time of irradiation and also by the time of perfusion (parameter [c]); EdU^*only*^ (i.e., BrdU^–^EdU^+^) cells (blue arrowheads) representing cells that were in S phase by the time of perfusion but not by the time of irradiation (parameter [d]). Two lower rows represent DCX-positive neurons. Gray dashed line outlines the inner border of SGZ. Scale bars show 100 μm.

Next, to assess whether exposure to gamma radiation affects S phase progression of progenitors 1 day after irradiation, we counted the number of EdU-labeled RGL and ANP cells (parameter [b] above) 2 h after the labeling, i.e., 24 h after irradiation (Figures 2C,D, 4 and Supplementary Table S1). A 1 Gy dose resulted in 71% decrease in labeled RGLs (*p* = 0.0003) and 63% reduction in labeled ANPs (*p* < 0.0001), and 5 Gy irradiation reduced the number of EdU-labeled RGLs by 97% (*p* < 0.0001) and of EdU-labeled ANPs by 96% (*p* < 0.0001). Using ANOVA for both RGLs and ANPs, we found large main effects for irradiation, with both RGL and ANP cells showing a significantly decreased ability to enter the S phase ∼6–22 h (given 1 h label bioavailability and 8–10 h S-phase length) after exposure to 1 Gy of gamma radiation, this ability being virtually abolished after the 5 Gy dose. Two-way ANOVA showed interaction between cell type and dose factors [*F*(2,10) = 167, *p* < 0.0001]. Also note that whereas in the control group the number of EdU^+^ RGL was the same as the number of BrdU^+^ RGLs (197 ± 55 vs. 201 ± 63), the number of EdU^+^ ANPs in that group was close to a half of the BrdU^+^ ANPs (1672 ± 131 vs. 2998 ± 394). Since the time between the labels’ injections was close to a full cell cycle, including duplication of proliferating cells, these numbers conceivably reflect the predominantly asymmetric mode of the RGL division and the symmetric mode of the ANP divisions.

We next examined whether the surviving BrdU-labeled RGLs and ANPs sustained their cell cycle progression ability. To determine whether cells that were in the S phase by the time of irradiation were capable of reentering the S phase 22–24 h later, we analyzed the number of cells that were labeled with both BrdU and EdU (parameter [c] above) (Figures 2E,F, 4 and Supplementary Table S1). One gray of gamma rays induced 81% reduction in BrdU^+^EdU^+^ double-labeled RGLs (*p* = 0.01) and 70% reduction in BrdU^+^EdU^+^ ANPs (*p* = 0.0002), compared to the control. If considered as a fraction of S phase cells that had been in the S phase 22 h before (i.e., BrdU^+^EdU^+^ cells as a fraction of BrdU^+^ cells), exposure to 1 Gy reduced the reentry of RGLs by 61% and of ANPs by 48% compared to the control. Similarly, if considered as a fraction of cells currently in S phase (i.e., BrdU^+^EdU^+^ cells as a fraction of EdU^+^ cells), 1 Gy reduced the fraction of previously cycling RGLs by 35% and of ANPs by 20% compared to the control. Neither BrdU^+^EdU^+^ RGLs nor BrdU^+^EdU^+^ ANPs were detectable after the 5 Gy dose. These results indicate that RGLs and ANPs that were undergoing DNA synthesis by the time of irradiation and have survived had a greatly reduced rate of cell cycle reentry 22 h after exposure to 1 Gy and lost the reentry ability after the 5 Gy dose.

Finally, we determined the changes in progenitors labeled only with EdU (i.e., BrdU^-^EdU^+^ or EdU^*only*^ cells) (parameter [d] above) as a measure of cells that were not in the S phase by the time of BrdU injection (whether fully quiescent, or traversing the cell cycle, but not the S phase, at that time). The number of EdU^*only*^ RGLs compared to the control group was decreased by 64% (*p* = 0.0002) and of EdU^*only*^ ANPs by 56% (*p* < 0.0001) after 1 Gy. Five grays of radiation reduced EdU^*only*^ RGLs by 95% (*p* < 0.0001) and EdU^*only*^ ANPs by 92% (*p* < 0.0001), with ANOVA showing large effects of irradiation for EdU^*only*^-labeled RGLs and ANPs (Figures 2G,H, 4 and Supplementary Table S1) and two-way ANOVA showing interaction between cell type and dose factors [*F*(2,10) = 126, *p* < 0.0001]. These results indicate that even those cells that were not in the S phase at the time of radiation exposure suffered profound impairment of the cell cycle progression that lasted for at least 22 h. Taken together, these results suggest that both dividing RGLs and ANPs are sensitive to 1 and 5 Gy of gamma rays. RGLs and ANPs that were in the S phase by the time of exposure and survived were significantly less likely to reenter the cell cycle 6–22 h later. In addition, gamma radiation negatively affected the overall level of RGL and ANP proliferation: even cells not in the S phase at the time of exposure (i.e., EdU^*only*^ cells) were less able to divide 24 h after exposure to 1 Gy and were virtually stalled 24 h after exposure to 5 Gy.

### Delayed Effects of Radiation on Neural Progenitors: 2 Months Post-exposure

**FIGURE 5:**
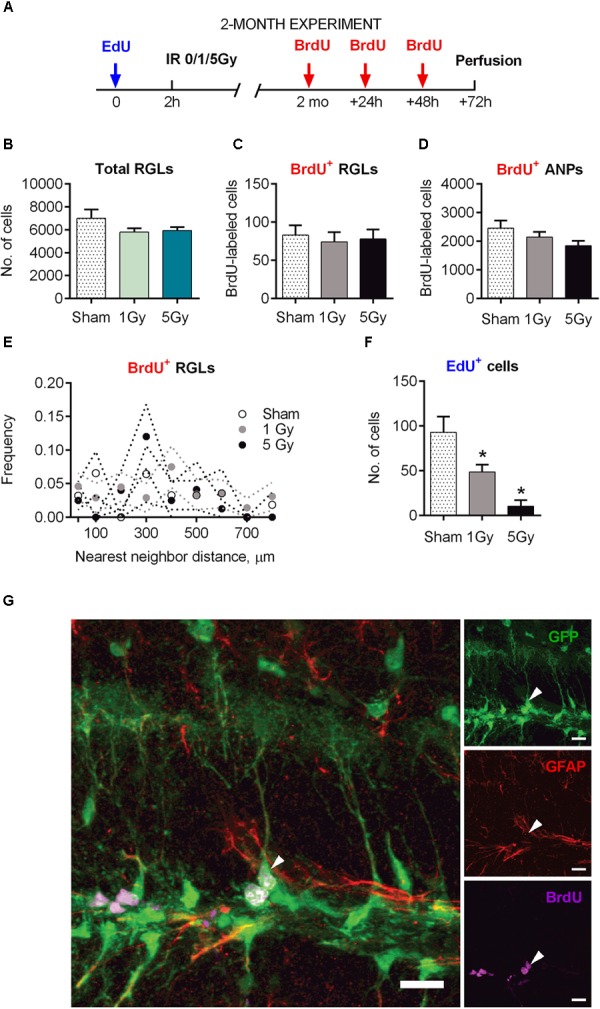
Maintenance of early and late progenitor cells at 2 months post-exposure to 0, 1, or 5 Gy (2-month experiment). **(A)** Experimental design. **(B)** Total RGLs (dividing and non-dividing). **(C)** Dividing (BrdU^+^) RGLs. **(D)** Dividing (BrdU^+^) ANPs. **(E)** Frequencies of nearest neighbor distances between BrdU-labeled RGLs. The frequencies were estimated as the number of cell pairs at particular distance bins per total number of BrdU^+^ RGLs. The dots and dotted lines represent mean frequencies and SEM ranges. **(F)** Survival of cells labeled with EdU at 2 h before the exposure. ^∗^*p* < 0.05, a comparison with sham group, Dunnett’s multiple comparison test after one-way ANOVA, multiple *t*-tests with Holm–Sidak multiple comparison method for EdU^+^ cells in **(F)** (see Supplementary Table S2 for detailed statistics). *N* = 7 mice were used in 0 Gy group, *n* = 9 in 1 Gy group, and *n* = 7 in 5 Gy group. Bars show means and standard errors. **(G)** Examples of a labeled RGL (arrowhead) surrounded by ANPs. Scale bar shows 20 μm.

Most of the RGL cells survived gamma irradiation (Figure 1B) and thus were potentially able to reconstitute neurogenesis. Still, such regeneration may be counteracted by sustained inflammation and vascular damage in the stem cell niche or irradiation-induced accelerated depletion of the stem cell pool. To examine the delayed effects of exposure to gamma rays, we analyzed subclasses of stem and progenitor cells in the DG 2 months after irradiation, with labels injected before (for EdU) and after (for BrdU) irradiation (Figure 5A).

The total number of RGL cells decreased by 32% in control mice 2 months after sham-irradiation, reflecting the expected overall decrease in DG stem cells from 1.5 to 5.5 months of age (Figures 1B, 5B). The total pool of RGL cells in mice that experienced radiation did not differ significantly from that in sham-irradiated controls 2 months after exposure to 1 (17% decrease, *p* = 0.15) or 5 Gy (15% decrease, *p* = 0.23). To assess whether the rate of progenitors’ division was affected by preceding irradiation, we labeled dividing cells by injection of BrdU 2 months after irradiation and analyzed the brains 24 h later, finding that the numbers of labeled cells in all three groups (0, 1, and 5 Gy) did not differ for RGL or for ANP (Figures 5C,D,G and Supplementary Table S2). In agreement with the data above on the total number of RGL cells, the fraction of dividing RGLs among all RGL cells did not change significantly: 1.3 ± 0.7% for 0 Gy, 1.3 ± 0.6% for 1 Gy, and 1.3 ± 0.5% for 5 Gy. Together, these results indicate that the rate of stem cell division in the DGs of irradiated mice returned to normal 2 months after irradiation.

Still, even if the overall number of stem cells and dividing stem cells was restored, their mode of division (asymmetric vs. symmetric) may have been affected by radiation and the subsequent recovery period, perhaps compensating for the loss of progenitors. Therefore, we next asked whether the rate of symmetric division of stem cells was modified by irradiation. We reasoned that symmetric divisions of RGLs would alter the spatial distribution of BrdU-labeled RGLs since such cells would generate pairs of closely positioned cells within the sectioned DG volume (Mineyeva O. et al., 2018; Mineyeva O.A. et al., 2018). Such pairs can be generated by chance, through cell cycle activation of unrelated adjacent stem cells, or as a consequence of symmetrical division of a common mother RGL cell. Therefore, by comparing labeled cell pairs’ frequency with and without irradiation, we could indirectly assess cell division mode. Toward this goal, we examined the frequencies of nearest-neighbor pairs generated by BrdU-labeled stem cells in each brain section. We determined the *xyz* positions of all BrdU-labeled RGLs and analyzed their pairs at <30 μm distances and at 0–800 μm distances with 100 μm bins in the control and irradiated groups. We found close pairs of BrdU-labeled RGLs only rarely, detecting only two such pairs (with 5 and 30 μm distance between the cells), in two out of seven brains in the control group; five such pairs in the 1 Gy-treated group of nine animals (three mice had one pair each with 28, 6, and 5 μm distance, and one animal had two pairs with 27 and 24 μm distance); and one such pair with 16 μm distance in the 5 Gy-treated group of seven animals. The frequencies of these and other pairs were not different from control (Figure 5E, *p* > 0.05 for all bins, *t*-test comparison of 1 or 5 Gy animals with controls for each bin, Holm–Sidak correction for multiple comparisons with *α* = 0.05). These results indicate that 2 months after their proliferation was disrupted by gamma irradiation, the spatial order of quiescent cells’ activation did not change, suggesting that RGLs predominantly preserved an asymmetric mode of cell division.

### Delayed Effects of Radiation on Neurogenesis: 2 Months Post-exposure

Even if the rate of progenitor division was restored, the path from stem cells toward differentiated neurons may have been altered by radiation exposure. To study this possibility, we first examined whether the overall extent of neuronal differentiation was affected by irradiation. In experiments where the label was injected 2 h before irradiation and animals were analyzed 2 months later, we found that the number of EdU-labeled cells (which mainly reflected the number of differentiated neurons born by the time of irradiation) was significantly reduced, with 60% reduction (*p* = 0.04) for 1 Gy and 92% reduction (*p* = 0.008) for 5 Gy (Figure 5F). This reduction in labeled cells was comparable to the losses in progenitors observed 24 h after irradiation, indicating that neuronal progenitors that survived radiation exposure did not compensate for the lost cells during the 2-month post-irradiation period.

**FIGURE 6:**
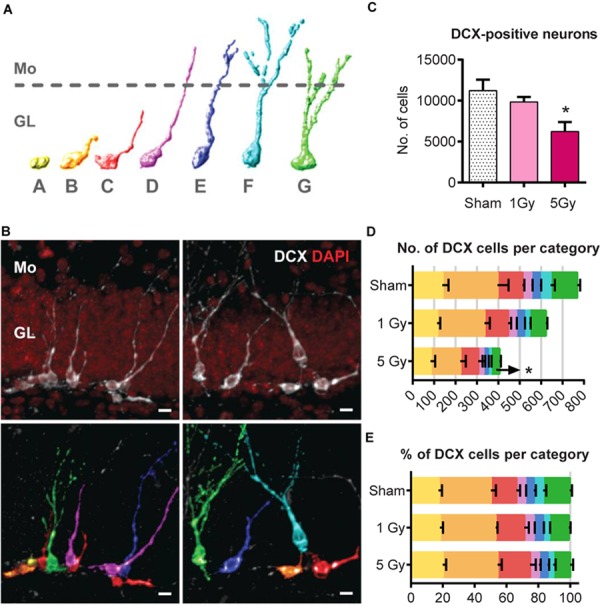
Generation of new neurons at 2 months post-exposure to 0 or 5 Gy (2-month experiment – scheme in Figure 5A). **(A)** Morphological categories of DCX cells. *A* category cells do not possess processes; *B* category cells have a short process that is no longer than a cell body; *C* category cells have a longer dendrite within the granule cell layer; *D* category cells extend their dendrite into the molecular layer but do not possess branching; *E* category cells have one branching point; *F* category cells have more than one branching node in the molecular layer; category *G* cells branch directly in the granular layer. Upper border of the granule cell layer is schematically shown with a dotted line. The same color scale defining the cell categories is used in **(B,D,E)**. **(B)** Examples of categorized DCX cells. DCX cells in original images (upper row) classified and colored according to the color scheme in **(A)** (lower row). Scale bar shows 10 μm. **(C)** All DCX-positive neurons. ^∗^*p* < 0.05, a comparison with sham group, Dunnett’s multiple comparisons after one-way ANOVA. *N* = 7 mice were used in 0 Gy group, *n* = 9 in 1 Gy group, and *n* = 7 in 5 Gy group. **(D)** Absolute numbers of DCX-positive neurons of each category. ^∗^*p* < 0.05, multiple *t*-tests with Holm–Sidak method (see Supplementary Table S2 for detailed statistics). **(E)** Fractions of DCX-positive neurons of each cell category. For **(D,E)**, *N* = 5 mice were randomly selected in each group.

Next, we analyzed the long-term effect of irradiation on the differentiation cascade that leads from early neuronal progenitors to fully differentiated neurons (Figure 6). To examine the impact of radiation on the population of young neurons 2 months post-exposure, we determined the number of cells expressing DCX, an early marker of neuronal lineage (Figure 6C and Supplementary Table S2). We found that irradiation with 1 Gy did not have a significant effect on the pool of DCX^+^ cells (12% reduction, *p* = 0.58); however, 5 Gy led to a 45% reduction (*p* = 0.013) in DCX^+^ cells.

Since DCX cells represent a mixture of progenitor subpopulations at different stages of neuronal maturation, we asked whether the two-fold decrease in DCX+ cells is unevenly distributed among those subpopulations, and examined morphologically distinct types of DCX-positive cells in a *post hoc* analysis. We defined early subclasses of neuronal progenitors (A–E-type cells, carrying a single apical process) as described (Plümpe et al., 2006) and categorized more mature cells with delicate apical dendritic trees as F and G types as described (Klempin et al., 2011; Figures 6A,B). Cells of the F category had a process branching with more than 1 node in the molecular layer, and G-category cells branched right in the granular layer. Even though identification of cells with processes can be potentially compromised by dendritic tree truncation upon sectioning, the G-category cells, which represented most mature DCX young neurons, had the most unambiguous morphology and could thus be reliably identified and enumerated (Klempin et al., 2011).

We compared the absolute (Figure 6D) and relative counts (Figure 6E) of each category cells for the control and irradiated groups. Exposure to 1 Gy did not affect any of the cell types (*t*-tests for sham vs. 1 Gy with correction for multiple comparisons using the Holm–Sidak method, with *α* = 0.05). However, irradiation with 5 Gy decreased the absolute number of G-category cells by 67% (*p* = 0.0004, with Holm–Sidak correction, with *α* = 0.05); differences in other categories, as well as the fractions of each category among all DCX cells, did not reach statistical significance. As the proportions remained unchanged, while G-category decreased, it is possible that less mature cell types were also affected. Together, our results indicate that while the total pool of stem cells was unchanged by gamma radiation 2 months after irradiation, the pool of DCX young neurons was significantly decreased.

### Delayed Effects of Radiation: 6 Months Post-exposure

**FIGURE 7:**
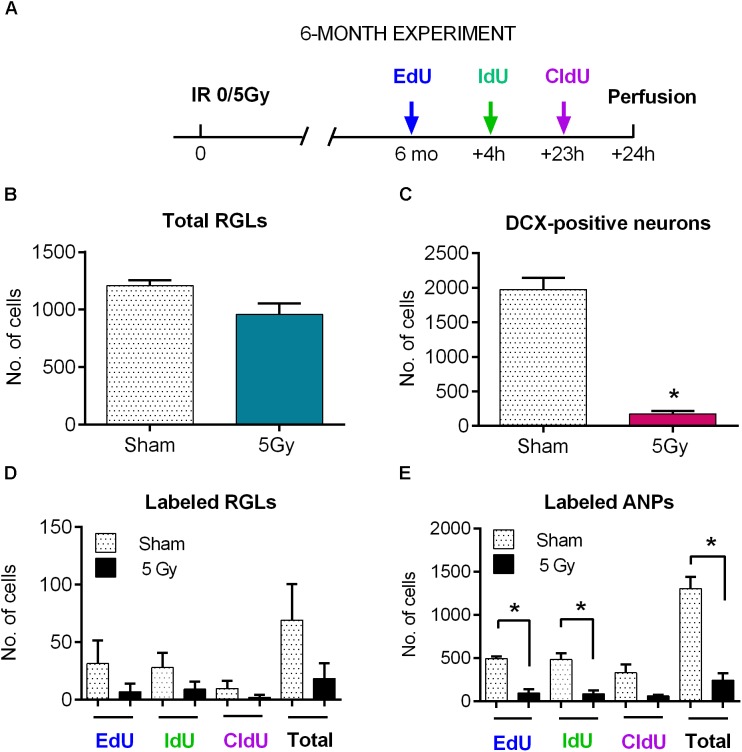
Maintenance of progenitor cells at 6 months post-exposure to 0 or 5 Gy (6-month experiment). **(A)** Experimental design. **(B)** Total RGLs (dividing and non-dividing). **(C)** Dividing (EdU, IdU, and CldU) RGLs. Right bars correspond to the total number of labeled cells. **(D)** Dividing (EdU, IdU, and CldU) ANPs. Right bars correspond to the total number of labeled cells. ^∗^*p* < 0.05, a comparison with sham group, *t*-test. See Supplementary Table S3 for detailed statistics. *N* = 4 mice were used in 0 Gy group and *n* = 4 in 5 Gy group. Bars show means and standard errors. Examples of labeled cells are shown in Figure 8.

**FIGURE 8:**
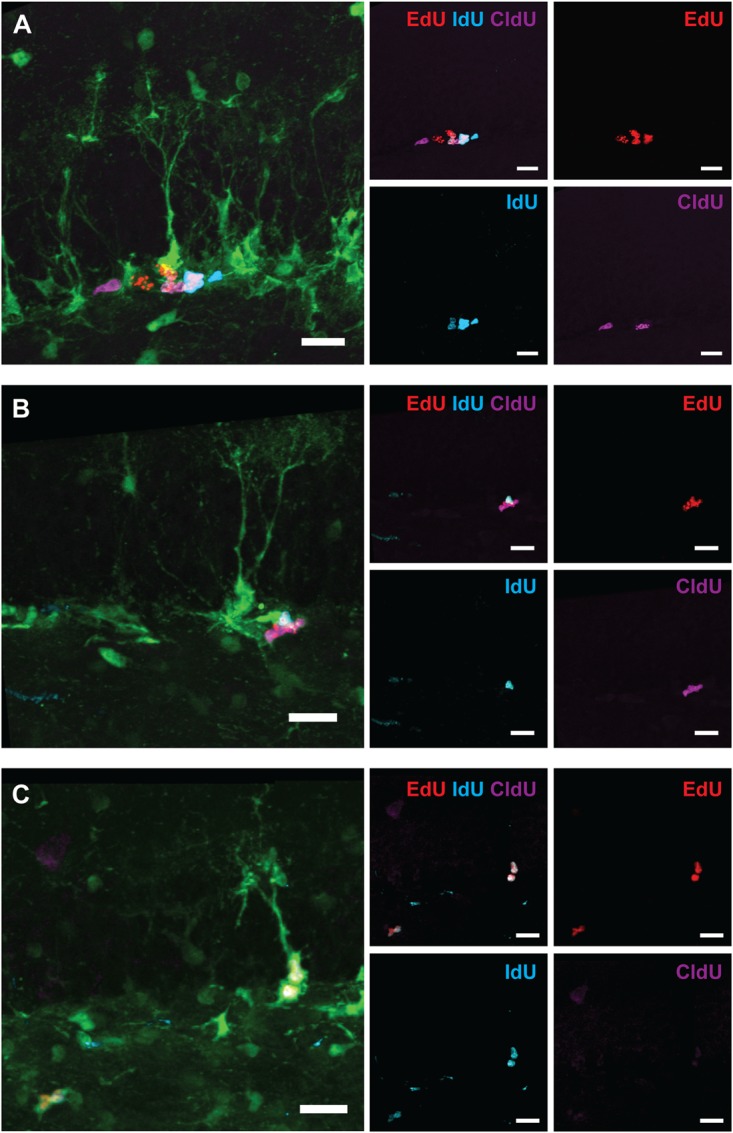
Examples of labeled cells analyzed in Figure 7 (6-month experiment – scheme in Figure 7A). **(A)** EdU^*only*^, IdU^*only*^, CldU^*only*^, and EdU^+^IdU^+^ labeled ANPs. **(B)** IdU^+^EdU^+^ and CldU^+^EdU^+^ labeled ANPs. **(C)** IdU^+^EdU^+^ labeled RGL and ANPs. Scale bar shows 20 μm.

Restored stem and progenitor cell division and partially reconstituted production of new neurons at 2 months could later progress to full recovery of neurogenesis from the spared reserve of quiescent neural stem cells and reconstituted pool of dividing neural stem cells. However, such recovery may be potentially compromised by aging, which could disproportionally affect irradiated stem cells and their microenvironment by reducing the number of RGL and ANP cells or of their dividing subsets, diminishing their propensity to produce new neurons, or decreasing the survival of their neuronal progeny. We therefore analyzed stem cells and early and late progenitors at 6 months post-irradiation, focusing on the 5 Gy dose because of its pronounced effects on neurogenesis (Figure 7A and Supplementary Table S3). To determine the changes in cell division and cell cycle reentry, we employed our new method for triple S phase labeling of dividing stem cells (Podgorny et al., 2018b) after injecting EdU, IdU, and CldU 24, 20, and 1 h before analysis, respectively (Figures 7A, 8).

The entire pool of RGL cells was not significantly affected 6 months after gamma rays exposure (Figure 7B, 21% decrease, *p* = 0.07). However, neurogenesis, as reflected in the number of DCX cells, was greatly diminished in 5 Gy-irradiated animals (Figure 7C, 91% decrease, *p* = 0.001). To further analyze changes in cell division, we compared the number of cells labeled with each nucleotide and the total number of labeled cells (Figures 7D,E and Supplementary Table S3). Even though the difference in the numbers of dividing RGLs in irradiated and control mice was profound (78% decrease for EdU-labeled RGL, 68% for IdU-labeled RGL, 80% for CldU-labeled RGL, and 74% for all labeled RGL, Figure 7D), it did not reach significance, mainly because of the drastically decreased numbers of dividing RGLs at that age (9–10 months). The number of ANPs labeled with EdU, IdU, or CldU was decreased in irradiated animals below the respective levels in the control group (Figure 7E and Supplementary Table S3, 81% decrease for EdU-labeled, *p* = 0.0005; 82% decrease for IdU-labeled, *p* = 0.006; 81% for CldU-labeled, *p* = 0.06; and 81% for total labeled, *p* = 0.0005).

To analyze changes in cell cycle reentry, we determined the fraction of EdU^+^CldU^+^ cells that reentered the cycle, comprising the cells that reentered the cell cycle and that exited the cycle (EdU/CldU^*only*^ and EdU^*only*^ cells, respectively) (Podgorny et al., 2018b). We did not detect RGLs reentering the cell cycle in either group (Supplementary Table S3); note, however, that the number of dividing RGL cells at the tested age was very low. However, we identified ANPs that reentered the cell cycle, their fraction being similar in both groups. These results indicate that the recovery observed at 2 months post-irradiation was not fully sustained by 6 months and suggest that even though the total pool of RGL cells remained unchained 6 months after irradiation, neurogenesis was greatly impaired at the level of stem cell/early progenitor proliferation.

## Discussion

Although the effects of gamma radiation on neurogenesis have been thoroughly investigated, inquiry into the response of the quiescent, dividing, and differentiating subpopulations of stem and progenitor cells has been limited by the available methods of cell cycle analysis. Here, we apply a new multiple-labeling/progenitor phenotyping technique to uncover short- and long-term consequences of exposure to gamma radiation on hippocampal neural stem cells and neurogenesis.

We found that while large part of cycling stem (RGL) and progenitor (ANP) cells did not survive subsequent irradiation, the total pool of quiescent stem cells, the majority of which were not in the cell cycle at the time of irradiation, did not decrease in number (Figure 1B). As expected, this was not the case for ANP, the majority of which are engaged in proliferation at any time. Our results indicate differential sensitivity of quiescent and dividing stem cells and of progenitor cells to gamma rays. These results also suggest that surviving stem cells may be able to effectively repopulate the neurogenic niche (Figure 4).

Remarkably, we found that proliferating RGL stem cells demonstrated higher resistance to the effects of gamma rays than their amplifying progeny (Figure 2). This emphasizes the distinct sensitivity of stem cells and their dividing and differentiating progeny and is in line with the notion of a heightened resistance of tissue-specific stem cells to genotoxic stress (Mohrin et al., 2010; Sotiropoulou et al., 2010; Insinga et al., 2013; Walter et al., 2015; Li et al., 2016).

Furthermore, we found that irradiation suppresses reentry of both cycling stem and progenitor populations (BrdU^+^EdU^+^ populations of RGL and ANP; Figures 2E,F) into the next cell cycle 6–22 h later. This suppression may be due to intrinsic mechanisms that slow down progression of the activated stem and progenitor cells in response to irradiation, or may reflect an altered stem cell niche environment, which inhibits cell division. These possibilities are supported by our finding that exposure to radiation suppresses division of neural stem and progenitor cells in the hippocampus 1 day after the event (EdU^+^ populations of RGL and ANP, Figure 2). They are also supported by the observation that even the cells that entered the division cycle for the first time or at least were not involved in DNA replication at the time of irradiation (EdU^*only*^ populations of RGL and ANP) were also suppressed in their division capability 22 h after irradiation (Figures 2G,H), perhaps reflecting an altered niche that was not conducive to cell division.

Irradiation-imposed restrictions on division of stem cells were partially mitigated 2 months after radiation exposure, when division of the RGL and ANP cells was restored, as reflected in the rates of activation and cell cycle reentry of RGLs and the reentry and survival of ANPs (their numbers being expectedly diminished due to the age-related decrease in the stem cell pool), as well as in spatial distribution of dividing RGLs. Still, even as cell division in the stem cell niche appeared restored when probed 2 months post-irradiation, finer features of neuronal differentiation and maturation were still affected by irradiation. Indeed, we found that the number of cells labeled before irradiation (which largely correspond to newborn differentiated neurons) was greatly diminished, reflecting the scarcity of neuronal progenitors (Figure 4E). Furthermore, the number of DCX-positive young neurons was decreased twofold by the preceding exposure to 5 Gy irradiation (Figure 6C). Finally, we found that the most mature class of young neurons (G category) was still affected by the radiation 2 months after the exposure (Figures 6D,E).

These defects may reflect the wave of maturation, with the most mature cohort (i.e., neurons born 4–6 weeks after irradiation) still carrying the signs of a damaged niche, and the less mature cohorts, having been born later, being largely unscathed by the reconstituted niche (alternatively, survival of this particular G subclass of young neurons was affected for 2 months and perhaps beyond). Notably, the overall cascade of maturation and its timing did not show overt changes, even though it has been exposed to post-irradiation inflammatory process (Rola et al., 2004b, 2007; Zou et al., 2012; Acharya et al., 2015), as the ratios between different progenitors’ subclasses were preserved.

Despite the partial restoration of the neurogenic niche 2 months after irradiation, the niche became less productive at 6 months. While the pool of RGLs was largely preserved (Figure 7B), there was a profound decrease in dividing ANPs, which translated into a dramatic loss in newborn DCX neurons (Figures 7C,E). Note that tenfold drop in DCX progenitors may be an extension of the twofold drop observed at 2 months, i.e., possibly indicating a trend radiation-induced decreased neurogenesis with time. Such overall impediment to stem and progenitor cell proliferation and neurogenesis may indicate cell-intrinsic changes, a deteriorating neurogenic niche (e.g., due to augmented inflammation), which supports the survival of the pool of stem cells but is not conducive to their production of new neurons, or a combination of cell intrinsic and extrinsic effects. Overall, the decrease in the number of Dcx-progenitors and dividing progenitors may be due to altered rates of stem cell recruitment, stem cell division, division of amplifying progenitors, or their survival at different stages of programmed elimination of neuronal progenitors.

In the intact brain, disposal of quiescent RGLs follows their activation and division (Encinas et al., 2011; Pilz et al., 2018), and the continuous diminishment of the RGL pool is accelerated by seizures or overstimulation (Sierra et al., 2015; Bao et al., 2017). Even if the majority of RGLs survive irradiation in the short term, their continued maintenance may potentially be affected by inflammation and DNA damage accumulated as the result of irradiation and aging. Interestingly, we found that the overall pool of RGLs was not reduced compared to aged-matched controls at 2 and 6 months post-exposure. This implies that the pools of intact and irradiated RGLs were being exhausted at the same rate. However, such preservation of the pool size does not guarantee that activation- or division-related features of RGLs are also preserved. Indeed, decreased production of new neurons 6 months after irradiation may be partially due to the decreased ability of stem cells to produce progeny.

Of note, we did not find indications of symmetric division of stem cells (Figure 5E), which could have been a conceivable strategy for a rapid restoration of the pool of dividing neuronal stem/progenitor cells after radiation-induced damage (although we cannot exclude the possibility that stem cells became briefly engaged in symmetric divisions shortly after irradiation).

Taken together, our results indicate that exposure to gamma radiation eliminates dividing neural stem cells (and, to a larger extent, progenitor cells), spares quiescent cells, and temporarily suppresses cell division within the neurogenic niche, with the division support by the niche largely restored 2 months later but deficient 6 months later. Note that most of these consequences of radiation exposure cannot be revealed using conventional labeling techniques but can be uncovered using the double- or triple-labeling/progenitor phenotyping approach applied here.

Our results on the gamma radiation effects on adult hippocampal neurogenesis raise several challenging questions. One relates to the differences between two neurogenic zone in the adult brain, the subventricular zone (SVZ) and the SGZ. In addition to a number of similarities, the response of SVZ to radiation shows better long-term recovery (Hellström et al., 2009; Daynac et al., 2016) and may show less distinction between quiescent and proliferating stem cells, instead being more specific to cell types (Barazzuol et al., 2017). While such differences between SVZ and SGZ may be largely region-specific, they may also reflect dissimilarities in the modes of maintenance and division of neural stem cells in those areas—limited renewal with predominantly asymmetric division in the SGZ (Encinas et al., 2011) vs. extensive self-renewal with symmetric divisions in the SVZ (Obernier et al., 2018), or differences in the response of the stem cell niches to irradiation (Hellström et al., 2011).

Another important question relates to the differences in how the developing and adult brain respond to radiation. For instance, the neonatal SVZ shows less proliferation arrest and more rapid recovery after X-ray irradiation than adult SVZ (Barazzuol et al., 2017). Note that the rate of stem cell division and neurogenesis is much higher in the neonatal than in the adult SVZ. It remains to be established whether such differences in the response to irradiation reflect differences in the efficiency of checkpoints or in repair mechanisms attendant to distinct division rates.

Furthermore, it is interesting whether the changes that we found after exposure to gamma rays are also observed after exposure to other types of radiation. Exposure to X-rays, gamma rays, or heavy ions leads to extensive perturbations in neural stem cells and neurogenesis (Monje et al., 2002; Rola et al., 2004a, 2005, 2008; Encinas et al., 2008; Hellström et al., 2009; Rivera et al., 2013; DeCarolis et al., 2014; Li et al., 2016; Barazzuol et al., 2017); note, however, that exposure to high-LET radiation may have a disproportionate effect on quiescent stem cells (Encinas et al., 2008; DeCarolis et al., 2014).

Yet another rising issue is the potential relevance of the results with neural stem cells to other types of stem cells, as related to their response to radiation. As in the adult brain, in several tissues stem cells are more resistant to radiation than more advanced progenitors. This observation is in line with the general notion of stem cells being more resistant to genotoxic stress (Mohrin et al., 2010; Sotiropoulou et al., 2010; Insinga et al., 2013; Walter et al., 2015; Li et al., 2016). The differential response of stem cells and their progeny may involve, among other mechanisms, stem cells’ reliance on non-homologous end-joining (NEHJ) as a preferred DNA repair mechanism during quiescence, and their entry into the cell cycle as the means of switching from NEHJ to more efficient modes of DNA repair (Mohrin et al., 2010).

Finally, our results point to a link between compromised neurogenesis and long-term cognitive impairments associated with irradiation. In particular, immature DCX-expressing neurons, which were produced at negligible numbers 6 months after irradiation, are critical for the acquisition of spatial learning, as well as reversal learning in rodents (Vukovic et al., 2013). Remarkably, the total pool of stem cells did not change significantly within the same time frame. This contrast between the preservation of stem cells and the diminishment of their DCX progeny is compatible with an impaired stem cell niche and raises a possibility that anti-inflammatory agents may be able to ameliorate radiation-induced cognitive dysfunctions.

Our findings on adult mouse hippocampal neurogenesis may have relevance for the radiation-exposed human nervous system. Evidence for neurogenesis persisting in the adult and old human brain is supported by several non-overlapping approaches (nucleotide incorporation, carbon dating, isolation of cells with stem potential, and post mortem immunocytochemistry (Eriksson et al., 1998; Roy et al., 2000; Spalding et al., 2005; Boldrini et al., 2009, 2012, 2014, 2018; Knoth et al., 2010; Spalding et al., 2013; Ernst et al., 2014; Kempermann et al., 2018), although the validity of post mortem immunocytochemistry data is currently debated (Boldrini et al., 2018; Kempermann et al., 2018; Sorrells et al., 2018). Importantly, all published reports support the notion of robust neurogenesis in children and young adolescents, which represent a large fraction of patients undergoing irradiation procedures in the clinic, underscoring the relevance of studying the impact of radiation on the hippocampal neurogenesis in the animal models.

In summary, our results indicate that gamma radiation hinders several steps of stem and progenitor cell division and neuron production. It will be a challenge to evaluate whether irradiation-induced changes in neurogenesis are also reflected in the connections that newborn neurons establish within and beyond the hippocampus.

## Materials and Methods

### Animals

For evaluating the effect of radiation on neural stem and progenitor cells we used heterozygous Nestin-GFP mice (Mignone et al., 2004) maintained on C57BL/6J background. Mice were housed from 4 to 10 per cage in a 12-h light–dark cycle with freely available food and water. This study was carried out in accordance with the recommendations of protocol #13-1, approved by Cold Spring Harbor Laboratory IACUC, and protocol 1, approved by Biomedical Research Committee, NRC “Kurchatov Institute.”

### Irradiation

Irradiation procedures were based on previously published protocols for whole-body and cranial gamma radiation that have used the power close to 1 Gy/min (Moravan et al., 2011; Corniola et al., 2012; Park et al., 2012; Acharya et al., 2015). For short-term experiment, a whole-body irradiation of 1.5-month-old mice was performed using ^137^Cs gamma rays source (Gammacell-40 Irradiator Nordion, Canada) with doses of 0 Gy (*n* = 4), 1 Gy (*n* = 5) for 1 min 4 s, or 5 Gy (*n* = 4) for 5 min 26 s. The background dose rate was 0.004 Rad/h in the irradiation chamber. Animals were placed together into the irradiation chamber, with the ^137^Cs source underneath the chamber. For the 2-month long experiment, whole-body irradiation of 3.5-month-old male mice was performed using ^60^Co gamma rays source (GUT-200M, NRC Kurchatov Institute, Moscow, Russia) with doses of 0 Gy (*n* = 7), 1 Gy (*n* = 9) for 1 min 10 s, or 5 Gy (*n* = 7) for 4 min 15 s. For the 6 month long experiment, whole-body irradiation of 3.5-month-old male mice was performed using ^60^Co gamma rays source (GUT-200M, NRC “Kurchatov Institute,” Moscow, Russia) with doses of 0 Gy (*n* = 4) or 5 Gy (*n* = 4) for 4 min 23 s. The background dose rate was 0.013 Rad/h. The treatment unit is certified by National Research Institute for Physical–Technical and Radio Engineering Measurements (Moscow, Russia) according to radiation dosimetry standards of State System for Ensuring Uniform Measurement. All animals in each group were irradiated simultaneously. Sham controls were treated the same as irradiated mice but without being exposed to radiation. No anesthesia was used before the irradiation procedures. After the treatment all animals were returned to their home cages for 24 h, 2 months, or 6 months, and kept under standard conditions.

### Administration of Thymidine Analogs and Perfusion

For the 24 h short-term experiment, BrdU (150 mg/kg, Sigma–Aldrich B5002) was administered intraperitoneally to all mice 2 h prior to the irradiation. Twenty-two hours after the treatment mice received an injection with an equimolar amount of EdU (123 mg/kg), then were anesthetized 2 h later (15% chloral hydrate, 10 ml/g of bodyweight) and perfused transcardially with 30 ml cold PBS and 50 ml of cold 4% PFA in PBS. For the 2 months long experiment, EdU (123 mg/kg, Invitrogen 10187) was injected 2 h prior to the irradiation. Two months later mice were injected with BrdU (150 mg/kg) three times, 24 h apart. Twenty-four hours after the last injection the animals were anesthetized and perfused. For the 6 months long experiment, thymidine analogs were injected 6 months after the irradiation: EdU (123 mg/kg, Invitrogen 10187) was injected 24 h prior to perfusion, IdU (178 mg/kg, Sigma I7125) was injected 4 h after EdU, and CldU (128 mg/kg, Sigma C6891) was injected 19 h after IdU. One hour after the last injection the animals were anesthetized and perfused.

### Immunohistochemistry

Following perfusion, brains were postfixed in 4% PFA overnight at 4°C, then transferred to PBS and kept at 4°C until sectioning. Vibratome sections from a randomly selected hemisphere were prepared in lateral-to-medial direction. The sections were collected in PBS and kept in PBS with sodium azide at 4°C or in cryoprotectant (1 volume of ethylene glycol, 1 volume of glycerin, and 2 volumes of PBS) at -20°C until staining. For staining, sections were first incubated with blocking and permeabilization solution (PBS containing 2% Triton-100X and 5% normal goat serum Abcam, ab7481) for 1 h at room temperature. The sections designated for the analysis of BrdU incorporation were treated before the immunostaining procedure with 2 N HCl for 30 min at 37°C and rinsed with PBS three times. Incubation with primary antibodies was performed in 0.2% Triton and 3% normal goat serum overnight at 4°C on shaker. After a thorough washing with PBS, sections were incubated with secondary antibodies in PBS for 2 h at room temperature in darkness on a shaker. After three washings with PBS, the sections were stained with EdU-click reaction and AlexaFluor 555 Azide, triethylammonium salt (Invitrogen A20012) according to Salic and Mitchison (2008). BrdU and EdU staining for the long-term experiment was performed on a separate set of sections. EdU, CldU, and IdU were stained simultaneously. A separate set of slices was stained with antibodies for DCX. After three last washings with 0.2% Triton in PBS and three washings with PBS, the sections were mounted on slides in PBS, slightly dried, and covered with coverglass after adding Fluorescent Mounting Medium (DAKO, S3023). Slides were dried overnight at room temperature, then transferred to 4°C and kept until imaging. The following antibodies and reagents were used for the 24 h experiment staining: rabbit anti-GFAP (DAKO Z0334) at 1:500 dilution and goat anti-rabbit AlexaFluor 405 (Invitrogen, A31556) at 1:500; chicken anti-GFP (Aves Laboratories GFP-1020) at 1:500 and goat anti-chicken AlexaFluor 488 (Invitrogen, A11039) at 1:500; azide AlexaFluor 555 for click reaction (Molecular Probes A20012) at 10 μM; mouse anti-BrdU (MoBU-1 clone Invitrogen, B35128) at 1:400 and goat anti-mouse AlexaFluor 633 (Invitrogen, A21052) at 1:500. The following antibodies and reagents were used for the 2 month experiment staining: chicken anti-GFP (Aves Laboratories GFP-1020) at 1:500 dilution and goat anti-chicken AlexaFluor 488 (Invitrogen, A11039) at 1:500; mouse anti-BrdU (MoBU-1 clone Invitrogen, B35128) at 1:400 and goat anti-mouse AlexaFluor 568 (Invitrogen, A11031) at 1:500; rabbit anti-GFAP (Invitrogen 180063) at 1:500 and goat anti-rabbit AlexaFluor 647 (Invitrogen, A21245); on separate sections, azide AlexaFluor 555 for click reaction (Molecular Probes A20012) at 10 μM; on separate sections, guinea pig anti-DCX (Millipore AB2253) and goat anti-guinea pig AlexaFluor 647 (Molecular probes A21450). The following antibodies and reagents were used for the 6-month experiment staining: chicken anti-GFP (Aves Laboratories GFP-1020) at 1:500 dilution and goat anti-chicken AlexaFluor 488 (Invitrogen, A11039) at 1:500; rabbit anti-GFAP (Invitrogen 180063) at 1:500 and goat anti-rabbit AlexaFluor 647 (Invitrogen, A21245); and guinea pig anti-DCX (Millipore AB2253) and goat anti-guinea pig AlexaFluor 647 (Molecular probes A21450). Staining for nucleotide analogs was performed as described in Podgorny et al. (2018b).

### Image Acquisition and Stereological Analysis

Quantitative analysis of cell populations was performed by means of design-based (assumption free, unbiased) stereology, as detailed in Encinas and Enikolopov (2008). Slices were collected using systematic-random sampling. One brain hemisphere was randomly selected per animal. The hemisphere was sliced sagittally, in a lateral-to-medial direction, from the beginning of the lateral ventricle to the midline, thus including the entire DG. The 50 μm slices were collected in six parallel sets, each slice 300 μm apart from the next. One set of eight to nine slices on average, covering the extent DG in the hippocampus, were used for cell counting. Six or seven sections were used for counting categorized DCX cells. Seven matching slices were analyzed for each animal (five animals per group) for quantification and categorization of DCX cells. All cells were counted under a 40× objective, excluding those in the uppermost focal plane, and DCX cells were counted with 20× objective for 2 month long experiment and with 40× for 6 month long experiment. The counts of cells from all slices were averaged, then normalized to the average number of sections from all animals, then multiplied by 6 and by 2 (the number of slices per animal and for two hemispheres), thus representing the total number of cells per two hippocampi. For total DCX cells (Figure 6C), the counts of cells from all slices were summed, then normalized to the average number of sections from all animals, then multiplied by 6 and by 2 (the number of slices per animal and for 2 hemispheres). For DCX cell categories (Figures 6A,B,D,E), the counts of cells from six to seven slices having the same morphology were summed and used in the analysis. The images for the short-term experiment were collected using an epifluorescence/bright field spinning disk confocal microscope UltraVIEW Vox (PerkinElmer) equipped with the Volocity 6.0.1 software suite (PerkinElmer). The images for the long-term experiment were collected using an epifluorescence/bright field spinning-disc microscope Andor Revolution WD (Andor) equipped with the iQ 3.1 software (Andor) or using laser scanning microscope FV1000 (Olympus). All images were imported in Imaris software (Bitplane) and counted manually.

### Statistical Analysis

For 1 day experiment analysis, each variable was assumed to be normally distributed. To gain power with less subjects, a minimum change of 30% was considered as minimally important, taking into account a large variability of these parameters observed in our previous experiments (SD up to 25%) for eight sections analyzed per animal on average. Before each comparison, Brown–Forsythe test was used to compare standard deviations of the means. BrdU/EdU-colabeled cells did not pass Brown–Forsythe test and were compared using *t*-tests (irradiated groups vs. Sham) without an assumption for equal SD, followed by a correction for multiple comparisons using Holm–Sidak method, with alpha 0.05. In cases when two-way ANOVA was used, cell type-corresponding counts passed Brown–Forsythe test for equal variances, and within-animal cell type values were matched for each animal. For total ANP counting, four animals were counted in group. For the data obtained in the 2 months long experiment, Kolmogorov–Smirnov test was used to test a normality of distributions. If appropriate, ANOVA was then performed. Dunnett’s test was used to compare every mean to a control mean, obtain 95% CIs of difference and correct for multiple comparisons with familywise error rate of 5%. EdU cells and DCX cells of each cell category were compared with *t*-tests (irradiated groups vs. Sham) without an assumption for equal SD, followed by a correction for multiple comparisons using Holm–Sidak method, with alpha 0.05. The same analysis was used for the comparison of cell pair frequencies at particular distances for 1 Gy vs. Sham groups and 5 Gy vs. Sham. Kolmogorov–Smirnov test was used to test normality of distributions. For DCX counting in 2 month long experiment, five animals were randomly selected from each group, and no animal was excluded. For the data obtained in the 6 months long experiment, a normal distribution was assumed, and *t*-test used for all variables. Statistical analysis and graph plotting were performed using Prism GraphPad version 6.04 for Windows (GraphPad Software^1^). No animal was excluded.

## Data Availability

The raw data supporting the conclusions of this manuscript will be made available by the authors, without undue reservation, to any qualified researcher.

## Author Contributions

OM and GE designed the experiments. OM, DB, AK, and AL performed the experiments. GE and KA provided funding. OM, DB, AK, AL, KA, and GE interpreted the results, made direct and intellectual contribution to the project, and approved the final version of the manuscript.

## Conflict of Interest Statement

The authors declare that the research was conducted in the absence of any commercial or financial relationships that could be construed as a potential conflict of interest.

## Footnotes

1 http://www.graphpad.com
