# Supplementary material for: Radiation Induces Distinct Changes in Defined Subpopulations of Neural Stem and Progenitor Cells in the Adult Hippocampus
Source: Front Neurosci. 2019 Jan 9;12:1013. doi: 10.3389/fnins.2018.01013 (PMC6333747; doi:10.3389/fnins.2018.01013)
Supplement: Supplementary file 1 [file Table_1.docx]

Supplementary Material

Radiation Induces Distinct Changes In Defined Subpopulations Of Neural Stem And Progenitor Cells In The Adult Hippocampus

Olga A. Mineyeva, Dmitri V. Bezriadnov, Alexander V. Kedrov, Alexander A. Lazutkin, Konstantin V. Anokhin, Grigori N. Enikolopov*

*** Correspondence:** Grigori N. Enikolopov: [grigori.enikolopov@stonybrook.edu](mailto:grigori.enikolopov@stonybrook.edu)

# Table S1. Acute effects of gamma-irradiation: 24 hr post-exposure

|  | **Cell type** | **Mean cells (SD)*** | | | **ANOVA** | **Sham vs. 1 Gy** | | **Sham vs. 5 Gy** | |
| --- | --- | --- | --- | --- | --- | --- | --- | --- | --- |
|  |  | **Sham** | **1 Gy** | **5 Gy** | **F and p values** | **Absolute 95%CI of diff.**** | **Relative 95% CI of diff. (% of Sham)** | **Absolute 95%CI of diff.**** | **Relative 95% CI of diff. (% of Sham)** |
| Survival of the stem cell pool | Total RGL cells | 10222 (645) | 9169 (1512) | 8443 (960) | F (2,10)=2.4 p=0.14 | -924 to 3029 p=0.33 | 9% incr. to 30% decr. | -304 to 3862, p=0.09 | 3% incr. to 38% decr. |
| Survival and proliferation of daughter progenitors | Total ANPs (GFAP-/ GFP+) | 25598 (7464) | 15341 (2946) | 9284 (1369) | F (2, 9)=12.3 p=0.0027 | 1569-18945 p=0.0235 | 6-74% decr. | 7625-25001 p=0.002 | 30-98% decr. |
| Survival of S/G2-IR cells (Parameter [a]) | BrdU RGLs | 201 (63) | 104 (53) | 54 (16) | F (2, 10)=9.4 p=0.0051 | 12-181 p=0.027 | 6-90% decr. | 58-236 p=0.003 | 29-117% decr. |
|  | BrdU ANPs | 2998 (394) | 1747 (385) | 193 (90) | F (2, 10)=72.9 p<0.0001 | 684-1818 p=0.0004 | 23-61% decr. | 2207-3402 p<0.0001 | 74-113% decr. |
| Proliferation (Parameter [b]) | EdU RGLs | 197 (55) | 58 (25) | 7 (8) | F (2,10)=33.3 p<0.0001 | 79-197 p=0.0003 | 40-100% decr. | 128-252 p<0.0001 | 65-128% decr. |
|  | EdU ANPs | 1672 (131) | 621 (138) | 70 (37) | F (2,10)=203 p<0.0001 | 854-1249 p<0.0001 | 51-75% decr. | 1394-1810 p<0.0001 | 83-108% decr. |
| Cell cycle reentering of S/G2-IR cells (Parameter [c]) | EdU/BrdU RGLs | 67 (32) | 13 (13) | 0 (0) | NA | ***mean diff. 54, SE of diff. 15, p=0.01 | 81% decr. SE of diff. 22% | ***mean diff. 67, SE of diff. 16, p=0.006 | 100% decr. SE of diff. 24% |
|  | EdU/BrdU ANPs | 811 (196) | 245 (155) | 0 (0) | NA | ***mean diff. 566, SE of diff. 117, p=0.002 | 70% decr. SE of diff. 14% | ***mean diff. 811, SE of diff. 98, p=0.0002 | 100% decr. SE of diff. 12% |
| Cell cycle progression of non-S-IR cells (Parameter [d]) | EdU-only RGLs | 129 (28) | 46 (18) | 7 (8) | F (2,10)=40.7 p<0.0001 | 50-117 p=0.0002 | 39-91% decr. | 87-158, p<0.0001 | 67-122% decr. |
|  | EdU-only ANPs | 861 (80) | 376 (65) | 70 (37) | F (2,10)=160 p<0.0001 | 377-595 p<0.0001 | 44-69% decr. | 677-906 p<0.0001 | 79-105% decr. |

* Estimated for the whole DG volume; ** Dunnett’s test for CIs and correction for multiple comparisons after one-way ANOVA, multiplicity adjusted p values; *** t-test for each irradiated group vs. Sham, without assuming equal SD, correction for multiple comparisons using the Holm-Sidak method, with alpha=0.05.

# Table S2. Long-term effects of gamma-irradiation: 2 months post-exposure

|  | **Cell type** | **Mean cells (SD)*** | | | **ANOVA** | **Sham vs. 1 Gy** | | **Sham vs. 5 Gy** | |
| --- | --- | --- | --- | --- | --- | --- | --- | --- | --- |
|  |  | **Sham** | **1 Gy** | **5 Gy** | **F and p values** | **Absolute 95%CI of diff.**** | **Relative 95% CI of diff. (% of Sham)** | **Absolute 95%CI of diff.**** | **Relative 95% CI of diff. (% of Sham)** |
| Survival of irradiated labeled cells | EdU-labeled cells | 121 (91) | 49 (24) | 10 (18) | NA | *** mean diff. 72, SE of diff. 31 p=0.04 | 60% decr. SE of diff. 26 | *** mean diff.111, SE of diff. 35 p=0.008 | 92% decr. SE of diff. 29 |
| Neurogenesis | DCX cells | 11218 (3018) | 9832 (1357) | 6218 (2604) | F(2,12)=5.6 p=0.02 | -2461-5234 p=0.6 | 22 incr. to 47% decr. | 1152-8848 p=0.01 | 10-79% decr. |
|  | G category cells* | 125 (29) | 77 (16) | 41 (15) | NA | *** mean diff. 48, t=3.2 df=8, SE of diff. 15 p=0.01, alpha>5% | 38% decr. SE of diff. 12% | *** mean diff. 84, SE of diff. 14, t=5.9 p=0.0004, alpha<5% | 67% decr. SE of diff. 11% |
| Proliferation | BrdU-labeled RGLs | 83 (34) | 74 (38) | 78 (33) | F(2,20)=0.12 p=0.89 | -34 to 51 p=0.84 | 41% incr. to 61% decr. | -40 to 50 p=0.95 | 48% incr. to 60% decr. |
|  | BrdU-labeled ANPs | 2451 (707) | 2143 (548) | 1843 (444) | F(2,20)=2.0 p=0.17 | -380 to 996 p=0.47 | 15% incr. to 41% decr. | -122 to 1337 p=0.11 | 5% incr. to 55% decr. |
| Fraction of dividing RGLs from total RGL pool | BrdU RGLs / total RGLs | 1.3 % (0.7) | 1.3 % (0.6) | 1.3% (0.5) | - | - | - | - | - |
| Survival of the stem cell pool | Total RGL cells | 6989 (2085) | 5790 (1029) | 5924 (784) | F(2,20)=1.7 p=0.21 | *** mean diff. 1199, SE of diff. 792, p=0.15 | 17% decr. SE of diff. 11 | *** mean diff. 1065, SE of diff. 842, p=0.23 | 15% decr. SE of diff. 12 |

* Estimated for the whole DG volume, ** Dunnett’s test for CIs and correction for multiple comparisons, multiplicity adjusted p values; *** t-tests for each irradiated group vs. Sham, without assuming equal SD, correction for multiple comparisons using the Holm-Sidak method, with alpha=0.05.

# Table S3. Long-term effects of gamma-irradiation: 6 months post-exposure

|  | **Cell type** | **Mean cells (SD)** | | **t-test*** | **Sham vs. 5 Gy** | **Sham vs. 5 Gy** |
| --- | --- | --- | --- | --- | --- | --- |
|  |  | **Sham** | **5 Gy** |  | **Absolute 95%CI of diff.** | **Relative 95% CI of diff. (% of Sham)** |
| Neurogenesis | DCX cells | 1974 (342) | 176 (80) | t=10.2 df=3.3 p=0.001 | 1269-2326 decr. | 9-117% decr. |
| Survival of the stem cell pool | Total RGL cells | 1210 (95) | 958 (192) | t=2.4 df=4.4 p=0.07 | 36 incr. to 540 decr. | %3 incr. to 44% decr. |
| 24-hr label-retaining | EdU-labeled RGLs | 32 (40) | 7 (14) | t=1.2 df=3.7 p=0.3168 | 36 incr. to 85 decr. | 113% incr. to 266% decr. |
|  | EdU-labeled ANPs | 491 (51) | 95 (85) | t=8.0 df=4.9 p=0.0005 | 268-524 decr. | 55% incr. to 107% decr. |
| 20-hr label-retaining | IdU-labeled RGLs | 28 (26) | 9 (13) | t=1.3 df=4.5 p=0.26 | 20 incr. to 57 decr. | 71% incr. to 204% decr. |
|  | IdU-labeled ANPs | 483 (143) | 88 (71) | t=5.0 df=4.4 p=0.0060 | 182-608 decr. | 38-126% decr. |
| 2-hr label-retaining | CldU-labeled RGLs | 10 (13) | 2 (5) | t=1.1 df=3.7 p=0.35 | 13 incr. to 28 decr. | 130% incr. to 280% decr. |
|  | CldU-labeled ANPs | 330 (190) | 61 (26) | t=2.8 df=3.1 p=0.06 | 30 incr. to 569 decr. | 9% incr. to 172% decr. |
| Total labeled RGLs | EdU+IdU+CldU | 69 (62) | 19 (26) | t=1.5 df=6 p=0.18 | 32 incr. to 134 decr. | 46% incr. to 194% decr. |
| Total labeled INPs | EdU+IdU+CldU | 1304 (133) | 244 (82) | t=6.8 df=6 p=0.0005 | 678-1443 decr. | 52-107% decr. |

*Unpaired t test with Welch's correction, two-tailed p value with alpha=0.05.
